# Identification by Automated Screening of a Small Molecule that Selectively Eliminates Neural Stem Cells Derived from hESCs but Not Dopamine Neurons

**DOI:** 10.1371/journal.pone.0007155

**Published:** 2009-09-23

**Authors:** Yi Han, Aaron Miller, Julie Mangada, Ying Liu, Andrzej Swistowski, Ming Zhan, Mahendra S. Rao, Xianmin Zeng

**Affiliations:** 1 Buck Institute for Aging Research, Novato, California, United States of America; 2 Invitrogen, Carlsbad, California, United States of America; 3 National Institute of Aging, Baltimore, Maryland, United States of America; Massachusetts Institute of Technology, United States of America

## Abstract

**Background:**

We have previously described fundamental differences in the biology of stem cells as compared to other dividing cell populations. We reasoned therefore that a differential screen using US Food and Drug Administration (FDA)-approved compounds may identify either selective survival factors or specific toxins and may be useful for the therapeutically-driven manufacturing of cells *in vitro* and possibly *in vivo*.

**Methodology/Principal Findings:**

In this study we report on optimized methods for feeder-free culture of hESCs and hESC-derived neural stem cells (NSCs) to facilitate automated screening. We show that we are able to measure ATP as an indicator of metabolic activity in an automated screening assay. With this optimized platform we screened a collection of FDA-approved drugs to identify compounds that have differential toxicity to hESCs and their neural derivatives. Nine compounds were identified to be specifically toxic for NSCs to a greater extent than for hESCs. Six of these initial hits were retested and verified by large-scale cell culture to determine dose-responsive NSC toxicity. One of the compounds retested, amiodarone HCL, was further tested for possible effects on postmitotic neurons, a likely target for transplant therapy. Amiodarone HCL was found to be selectively toxic to NSCs but not to differentiated neurons or glial cells. Treated and untreated NSCs and neurons were then interrogated with global gene expression analysis to explore the mechanisms of action of amiodarone HCl. The gene expression analysis suggests that activation of cell-type specific cationic channels may underlie the toxicity of the drug.

**Conclusions/Significance:**

In conclusion, we have developed a screening strategy that allows us to rapidly identify clinically approved drugs for use in a Chemistry, Manufacture and Control protocol that can be safely used to deplete unwanted contaminating precursor cells from a differentiated cell product. Our results also suggest that such a strategy is rich in the potential of identifying lineage specific reagents and provides additional evidence for the utility of stem cells in screening and discovery paradigms.

## Introduction

The ability to expand human embryonic stem cells (hESCs) unlimitedly in culture and to differentiate them into specific somatic cell types [1] make them a useful tool in the development of hESC-based automated screening platforms for drug discovery. Although this possibility has not yet attracted as much attention as the ideas of cell replacement, personalized medicine and other more direct clinical applications, hESCs are expected to be superior to most commonly used cell-culture models of drug discovery which employ tumor-derived or immortalized cell lines or primary cell culture. This is because tumor-derived and immortalized cells are often karyotypically abnormal and may diverge physiologically from normal cells in various respects, whereas primary cells have limited capacity for expansion.

To enable the development of hESC-based automated screening, it is essential that current limitations surrounding hESC culture be overcome. For example, hESCs and their differentiated derivatives must be cultured without feeder layers in a format that is amendable to automated screening such as 96 well plates. Unlike mouse embryonic stem cells which can be efficiently expanded and differentiated from single cells, hESCs are routinely passaged as small clumps of cells or differentiated *via* embryoid bodies formed from tens to hundreds of cells [1]. The recent advances in culturing hESCs in defined media [2], [3], [4] and the report of increased cloning efficiency of hESCs [5] make it possible to develop protocols for culturing hESCs in large numbers. Nevertheless, feeder-free culturing of hESCs is not a routine success. Excellent outcomes notwithstanding, hESC-based screening platforms are limited by the difficulties of generating homogeneous and lineage-specific differentiated populations from hESCs while culturing them in large numbers for prolonged periods.

Given our extensive experiences in neuronal differentiation of hESCs [6], [7], [8] and the potential application of hESC-derived neurons in cell replacement therapies for neurodegenerative diseases, we designed a set of experiments aimed at developing a hESC-based automated assay for screening small molecules that have differential toxicity to hESC-derived NSCs and their differentiated neural progenies. We reasoned that the development of this assay would help identify chemical compounds that may be useful for eliminating proliferating cells in potential hESC-derived cell therapy products. To this end, we chose to use the National Institute of Neurodegenerative Diseases and Stroke (NINDS) collection of FDA-approved drugs for assay optimization and pilot screening. The bioactivity of the compounds in this library and the ready availability of individual compounds identified as hits for follow-up studies make this library ideal for pilot screenings. Furthermore, these routinely used drugs have been highly optimized to hit specific targets and in nearly all cases the mechanisms of action are known.

By comparative screening on hESCs and hESC-derived homogenous NSCs using the NINDS collection, we were able to identify compounds that had differential toxicity to both cell populations. Hits obtained in the primary screen were then retested and a small subset was assayed for dose-responsiveness. One confirmed dose-responsive compound, amiodarone HCl, was further tested for toxicity in postmitotic neurons. We found amiodarone HCL to be toxic to NSCs but not to postmitotic neurons, indicating its potential use for depleting proliferating NSCs in hESC-derived cell populations for possible neural transplantation.

## Materials and Methods

### Culturing of hESCs and hESC-derived NSCs

hESC lines I6 and H9 were maintained on Matrigel (BD Biosciences, Bedford, MA; http://www.bdbiosciences.com)coated dishes in medium (comprised of Dulbecco's Modified Eagle's Medium/Ham's F12 supplemented with 20% knockout serum replacement (KSR), 2 mM non-essential amino acids, 4 mM L-glutamine, 0.1 mM β-mercaptoethanol, 50 µg/ml Penn-Strep, and 4 ng/ml of basic fibroblast growth factor) conditioned with mouse embryonic fibroblasts for 24 hours as previously described [9], [10].

To derive NSCs as previously described [11], hESC colonies were harvested using a scraper and cultured in suspension as EBs for 8 days in ESC medium minus FGF2. EBs were then cultured for additional 2–3 days in suspension in neural induction media containing DMEM/F12 with Glutamax, 1 xNEAA, 1 xN2 and FGF2 (20 ng/ml) prior to attachment on cell culture plates. Numerous neural rosettes were formed 2–3 days after adherent culture. To obtain a pure population of NSCs, rosettes were manually isolated and dissociated into single cells using Accutase. The NSCs population was expanded in Neurobasal media containing 1x NEAA, 1x L-Glutamine (2 mM), 1x B27, LIF and FGF2 20 ng/ml.

Dopaminergic neuronal differentiation of hESC-derived NSCs was induced by medium conditioned on the PA6 stromal cell line for 4 weeks [12]. The media contained GMEM with 10%KSR, 1x non-essential AA, 1x Na pyruvate and 1x b-mercaptoethanol and was harvested from the PA6 culture every 24 h for a period of 1 week.

Human astrocytes were purchased from Sciencell Research Laboratories (isolated from human cerebral cortex, Cat# 1800, Carlsbad, CA) and were cultured in human astrocyte medium (Sciencell, Cat# 1801) on poly-L-lysine coated tissue culture dishes. Media was changed every other day and cells were passaged once a week at a 1∶4 ratio.

2102Ep cells, derived from a primary human testicular teratocarcinoma and later subcloned [13] (ATCC) were grown on tissue culture dishes in medium containing DMEM supplemented with 2 mM Glutamax and 10% fetal bovine serum. Media was changed every day and cells were passaged every 3–4 days at a ratio of between 1∶4 to1∶6.

### Drug Treatment and ATP assay

hESCs and NSCs were passaged onto 96 well plates at a density of 5×10^4^ and 2.6×10^4^ cells respectively in 200 µl media and incubated at 37°C for 48 hours. Media was changed every day for hESCs and every other day for NSCs and additionally changed prior to drug treatment. The cells were treated with compounds from the NINDS library diluted in 100 µl of either ESC or NSC media to a final concentration of 2.5 µM in 0.01% DMSO. Cells were incubated in the presence of drug for an additional 48 hours at 37°C before assaying. For all sampling, ESC and NSC plates were processed in parallel for one drug or control condition at a time.

For ATP measurements, the media was removed, cells were washed 1x in milliQ water and reconstituted in 50 µL ATP-Lite Mammalian Lysis Buffer and shaken for 5 minutes. Two 10 µL aliquots of lysed cells were replated onto separate 96 well plates for later protein measurements.

For measuring the effect of TNFα on NSCs, I6 NSCs were passaged onto fibronectin-coated 4-well plates in Neurobasal media supplemented with 1X B27, 2 mM L-glutamine and 10 ng/ml of both bFGF and LIF growth factors. Cells were recovered for 12 hours at 37° and then either left untreated or treated with solTNFα at the concentrations indicated. Cultures were observed for 24 hours after solTNFα treatment for signs of cell death and imaged with microscopy.

### Immunocytochemistry

Immunocytochemistry and staining procedures were as described previously [14]. Briefly, hESCs at different stages of dopaminergic differentiation were fixed with 2% paraformaldehyde for half an hour. Fixed cells were blocked for one hour in 0.1% Triton X-100 PBS supplemented with 10% goat serum and 1% BSA, followed by incubation with the primary antibody at 4°C overnight in 0.1% Triton X-100 with 8% goat serum and 1% BSA. Appropriately coupled secondary antibodies (Molecular Probes) were used for single and double labeling. All secondary antibodies were tested for cross reactivity and non-specific binding. The following primary antibodies were used: Oct-4 (19857 Abcam) 1∶1000; β-III tubulin clone SDL.3D10 (T8660 Sigma) 1∶500; Nestin (611658 BD Transduction laboratories) 1∶500 and TH (P40101 Pel-Freez) 1∶500, and as secondary antibodies: Alexa Fluor 594 Goat Anti-Mouse, Alexa Fluor 488 Goat Anti-Rabbit, Alexa Fluor 594 Goat Anti-Rabbit. Hoechst 33342 (Molecular Probes H3570) 1∶5000 was used for nuclei identification. Images were captured on a Nikon fluorescence microscope.

### Microarray analysis using BeadArray platform

RNAs isolated from NSCs and neurons with and without drug treatments were hybridized to Illumina HumanRef-8 BeadChip (Illumina, Inc., San Diego, CA, performed by Microarray core facility at the Burnham Institute for Medical Research). The Illumina array data were normalized by the quantile method, and then transformed log2 ratio values for a zero mean for expression values of each gene across all samples. The statistical and bioinformatics analyses were conducted by using R and the bioconductor package (www.bioconductor.org). The gene set enrichment analysis was conducted using the GSEA software (www.broad.mit.edu/gsea).

## Results

### Culturing of multiple hESC and hESC-derived NSC lines in 96-well plates

We have previously shown that NSCs can be generated from multiple hESC lines and can be cultured for prolonged periods without losing their ability to differentiate into neurons, astrocytes and oligodendrocytes [11]. The hESC lines H9 and I6 and their NSC derivatives behave similarly in culture and were used for this study.

**Figure 1:**
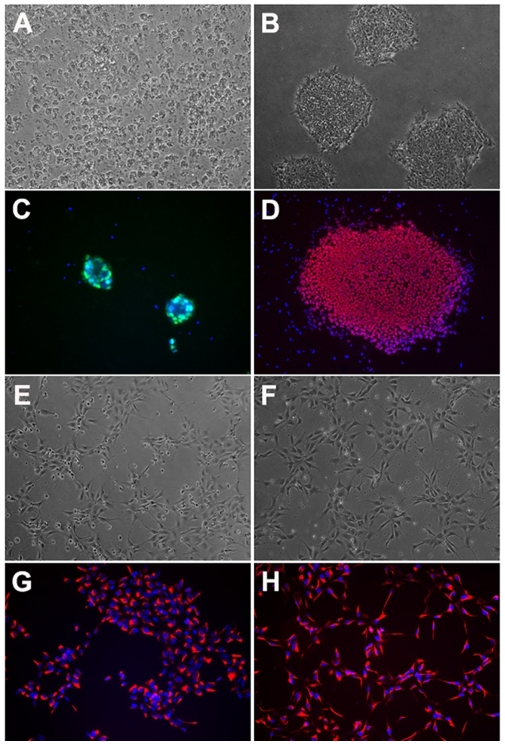
Morphology and expression of stem cell markers in hESCs and hESC-derived NSCs cultured in 96-well plates. (A–B) Typical undifferentiated hESC morphology 24 hours after plating (A) and 3 days after passaging (B). (C–D) Expression of pluripotent markers Oct4 in cells cultured in 96-well plates (C, Oct4 = green, nuclei = blue) and colonies cultured in 60 mm dishes (D, Oct4 = red, nuclei = blue). (E–F) Homogenous hESC-derived NSCs are morphologically similar whether cultured in 96 well plates (E) or larger 60 mm dishes (F). (G–H) Uniform expression of nestin in NSCs cultured in 96 well plates (G) and 60 mm dishes (H) Nestin = red, nuclei = blue.

For adapting to a 96-well format culture, hESCs were dissociated into single cells by Accutase. Tiny colonies were formed 24 h after plating (Fig. 1A) and typical undifferentiated hESC morphology was observed 2–3 days after passage (Fig. 1B). No differences in the expression of the pluripotent marker Oct4 (Fig. 1C–D) were found between cells cultured in 96-well plates and hESCs routinely passaged in medium conditioned on MEF in larger dishes (35-mm or 60-mm dishes). NSCs cultured in 96-well plates were morphologically indistinguishable from cells cultured in larger dishes (Fig. 1E–F) and uniformly expressed the NSC specific marker Nestin (Fig. 1G–H).

### Screening design, primary screening and retest of hits

To identify compounds that are toxic to hESCs, hESC-derived NSCs, or both, we screened 720 FDA-approved drugs of the NINDS collection by testing the toxicity of each drug at a dose of 2.5 µM. For endpoint measurement of cell death caused by drug toxicity, we used a widely accepted ATP assay that measures changes in ATP level as an indicator of cellular response to cell death. In this assay, total ATP content per well was measured and normalized to the total cellular protein.

**Figure 2:**
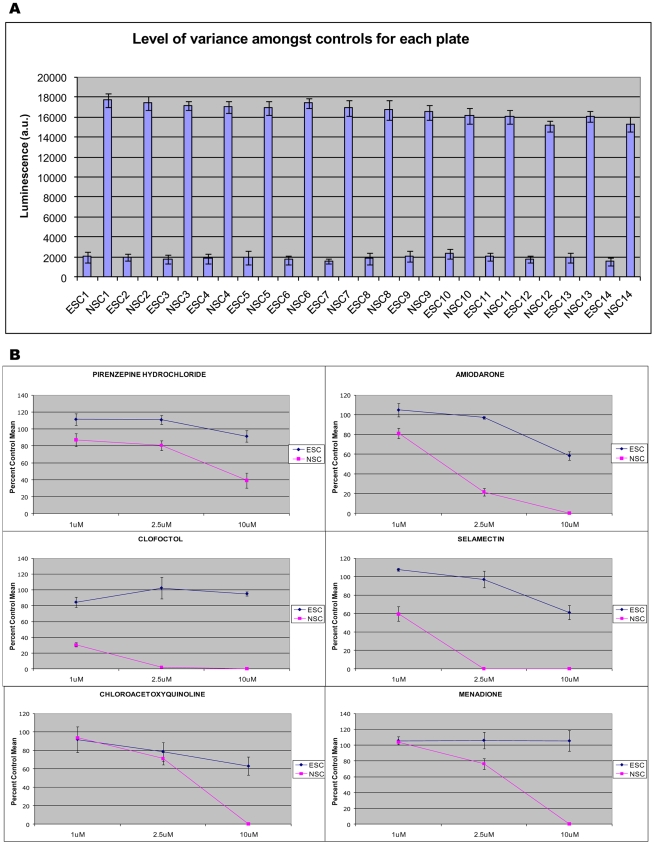
Primary screens and retests with the NINDS collection. (A) ATP levels in hESCs and NSCs. (B) Dose response of NSC and hESC to selectively screened NINDS compounds. Hits obtained in the primary screen were retested and validated to be toxic to NSCs in a dose-responsive manner.

In general, NSC-containing wells had much higher ATP levels than the hESC wells (Fig. 2A, standard deviation for variance in each plate provided in Supplementary Materials Table S4), consistent with recent reports that ATP levels are higher in differentiated EBs than in undifferentiated hESCs [15]. Hits were defined based on the ability of a compound to affect ATP levels relative to DMSO controls on each plate. Nine compounds, pirenzepine HCL, amiodarone HCL, selamectin, clofoctol, perhexilline maleate, griseofulvin, chloroactoxyquinoline, menadione and hexetidine were identified as “NSC Killers” in this primary screen. Application of these nine drugs reduced ATP concentrations with at least 2-fold or more potency for NSCs than hESCs, and NSC values were 15% or more below the control mean. In contrast, no compound was found to be specifically toxic to hESCs based on the same criteria.

We then retested the nine hits from the NINDS library screening in 96-well plates. Three concentrations of each compound (1 µM, 2.5 µM and 10 µM) were used in the retest. Six of the nine compounds, amiodarone HCL, selamectin, chloroacetoxyquinoline, menadione, pirenzepene and clofoctol showed a dose-dependent specific toxicity as demonstrated by reduced ATP concentrations in treated NSCs versus untreated NSCs, untreated hESCs and treated hESCs (Fig. 2B). Notably, of these 6 compounds that demonstrated dose responsive toxicity to NSCs, selamectin and amiodarone HCL had the most dramatic effect on NSC survival (Fig. 2B, p<0.001 for amiodarone HCL treated NSC versus similarly treated ESC, N = 3 independent replicates). Overall, these results indicate that changes in ATP levels are a reliable indicator of cell death in stem cell populations upon drug insults and may have utility for hESC-based automatic screening assays.

### Revalidation in larger numbers of cells and behavior of a candidate molecule on postmitotic neurons

**Figure 3:**
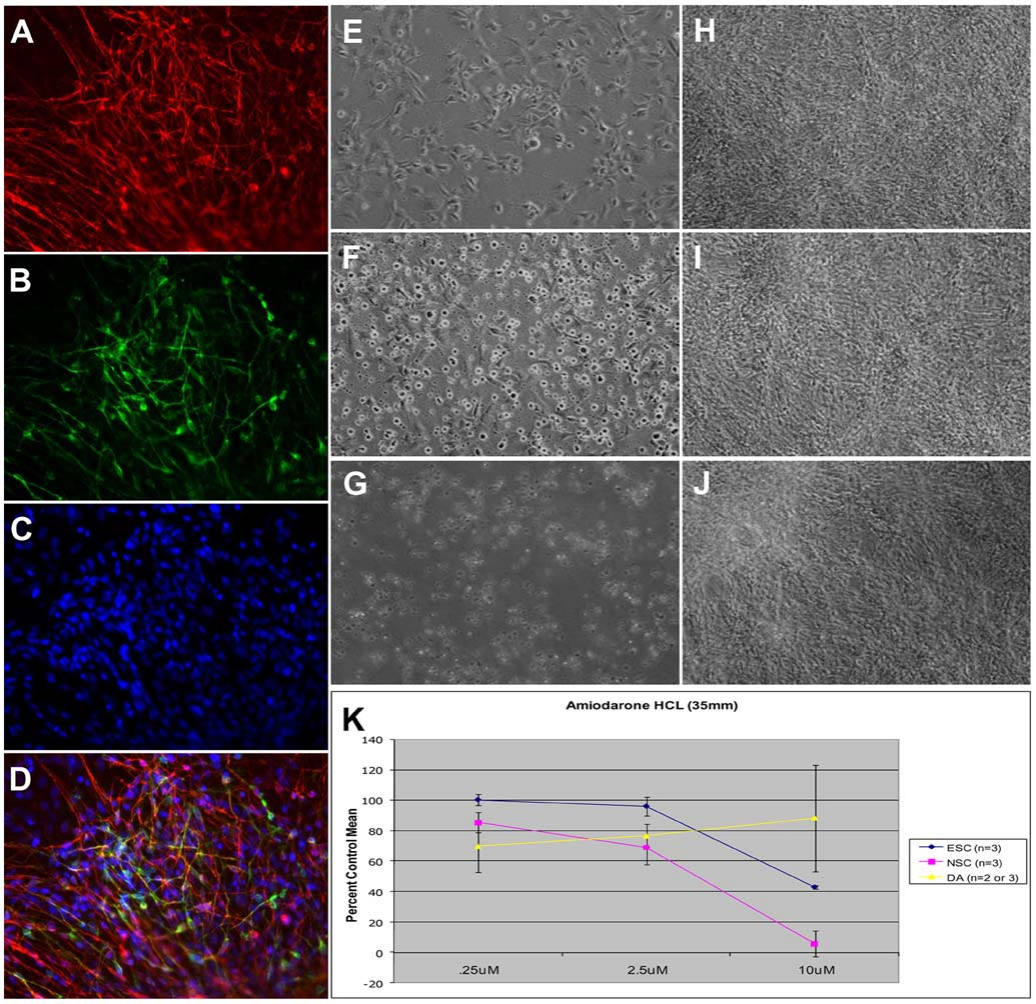
Validation of a compound in larger numbers of cells. (A–D) Expression of β-III tubulin (A, red) and TH (B, green) in NSCs that had been differentiated for 4 weeks (C = nuclei, blue, D = merge). (E–G) NSCs after 2 (E), 4 (F) and 8 (G) hours exposure to amiodarone HCl (H–J) dopaminergic neurons after 2 (H),4 (I) and 8 (J) hours exposure to amiodarone HCl. (K) Changes in ATP levels in hESC (blue line), NSC (pink line) and dopaminergic neurons (yellow line) after exposure to three doses of amiodarone HCl for 48 h (at 10 um NSC v DA p<0.001, N = 3 replicates)

For potential hESC-based neural replacement therapy, it would be useful to identify compounds that are selectively toxic to proliferating NSCs and not terminally differentiated postmitotic neurons. We therefore decided to interrogate the effects of one retested compound, amiodarone HCl, on NSCs and their differentiated derivatives. For postmitotic neurons, we chose to use an established neuronal differentiation culture system in which NSCs were induced to differentiate into dopaminergic neurons by medium conditioned on stromal cells for 4 weeks. After 4 weeks of differentiation, the majority of the cells (>60%) expressed the postmitotic neuronal marker β-III tubulin with a subset (about 50% of total neurons) additionally expressing TH, a marker for midbrain dopaminergic neurons (Fig. 3A–D). Less than 1% of the cells were positively stained for Sox1, a marker for NSCs (data not shown). Cells at this stage are referred to as dopaminergic neurons in this study.

NSCs and dopaminergic neurons grown in 35-mm dishes were exposed to amiodarone HCl. Cell death was observed in NSCs 2 hours after drug exposure, with more than 90% cell death evident by 8 hours (Fig. 3E–G). In contrast, no toxic effect was observed in dopaminergic neurons up to 8 hours after exposure to amiodarone HCl (Fig. 3H-J) at the highest dose (10 µM). At 10 µM, amiodarone HCL reduced ATP levels to less than 15% of the control mean specifically in the NSC population (Fig. 3K). In contrast, at this concentration amiodarone HCL was not toxic to dopaminergic neurons. Interestingly, the effect seen in hESC was intermediate between NSCs and dopaminergic neurons. To confirm the specificity of effect of amiodarone treatment on NSCs and rule out the possibility that the different media contributed to the protection seen for dopaminergic neurons, we derived neurons in defined media [11] and treated them with amiodarone HCL. Like neurons derived by PA6 conditioned medium, neurons generated in defined media were not susceptible to amiodarone toxicity (data not shown).

### Effects of amiodarone HCl on glia (non-neuronal) cells

**Figure 4:**
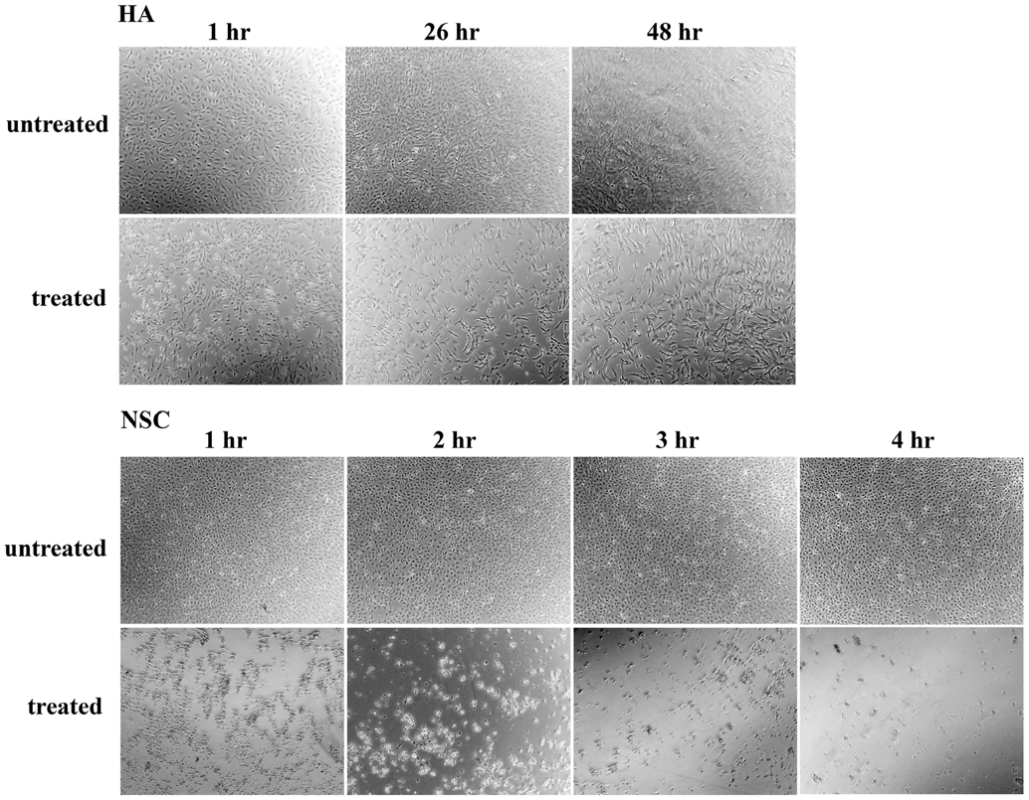
Effect of amiodarone HCl on glia cells. Human Astrocytes (HA, top panel) after 1, 26 and 48 hours exposure to amiodarone HCl and untreated cells. NSCs, either after 1, 2, 3 and 4 hours exposure to amiodarone HCl or left untreated are shown on the bottom panel for comparison.

To further confirm the specificity of amiodarone HCl 's toxicity on NSCs but not cells differentiated from NSCs, we tested the effect of amiodarone HCl on human fetal-derived astrocytes [16], a non-neuronal cell type in the nervous system. As seen in Fig. 4, amiodarone HCl did not cause astrocyte cell death up to 48 hours after treatment, whereas once again massive cell death occurred in similarly treated NSCs within one hour of drug administration. As an additional control we also tested the effect of amiodarone HCl on an immortal cell line 2102Ep cells [13]. Like terminally differentiated dopaminergic neurons and astrocytes, no effect was found on 2102Ep cells 48 hours after treatment (data not shown).

### Pathways activated by amiodarone HCl

In order to validate that the observed cell death was specific to the action of amiodarone HCL, and possibly dissect the mechanism of action of this compound, we performed a gene expression analysis of NSCs and postmitotic neurons receiving amiodarone HCL. Given that changes in gene expression profiles will likely be seen after a short period exposure to drugs, and that most cells had undergone cell death in as little as 8 hours (Fig. 3E–G), we compared gene expression of cells prior to and after 4 hours of exposure to the drugs. The dataset generated from the expression analysis, along with quality control data and the numbers of genes altered are provided in Supplementary Table 1 (Table S1) and Supplementary Fig. 1 (Figure S1).

**Table 1:** Ion channel gene expression in NSCs with and without amiodarone HCl treatment compared to similarly treated dopaminergic neurons.

Category	Gene	Treated NSC	Untreated NSC	Treated DA Neuron	Untreated DA Neuron
**H ion transporters**	**ATP6V1A**	1155.9	1093.8	2621.5	2441.8
	**ATP6V1B2**	385.4	331	724.8	438.2
	**ATP6V0D1**	3355	2760.4	2920.7	2452.5
	**ATP5B**	7030.3	5745.1	5217.1	4313.4
	**ATP6V0A2**	190.2	163	131.3	108
	**SLC2A11**	22.8	31.1	.9	34.9
	**SLC35B1**	1769.8	1380.4	1450.4	1122.8
	**SLC2A1**	1274.8	1574.5	87.1	86.7
**Amine transporters**	**SLC1A2**	15.7	21.3	809.7	681
	**SLC1A3**	552.1	433.5	2926	2578.1
	**SLC6A3**	−1.2	4.8	−3.4	10.5
	**SLC6A9**	329.2	251.5	36570.5	489.3
	**SLC6A12**	9.6	7.4	10.8	10.4
	**ATP1A1**	586.9	615.7	426.9	341.4
	**ATP1A1**	786.4	725.1	471	399.4
**Cl channels**	**CLCN6**	349.8	311.8	998.5	745.9
	**CLCN7**	1305.5	903.2	1261	1058.4
	**CLCN3**	566.5	440.4	541.2	497.2
	**CLCN2**	41.6	30.2	24	21.7
	**CLIC1**	122.2	94.9	22.9	18
**Voltage gated Na channels**	**SCN9A**	1.9	2.6	245.7	175.1
	**SCN1A**	−0.5	7.6	115.6	110.2
	**SCN3A**	5.7	6.3	60.4	72.3
**Amiloride sensitive Na channels**	**ACCN1**	16.5	15.4	387.4	360.1
	**ACCN3**	11	5.1	35.7	38.1
	**ACCN2**	185.7	167.4	862.7	837.1
**Rectifier K channels**	**KCND2**	2.3	8.9	269.9	225.5
	**KCNQ2**	138.1	137	1753.5	1378.2
	**KCNC4**	4.5	5.8	58.7	38.7
	**KCNJ4**	15.9	11.6	66.1	64.3
	**KCNQ3**	3.4	5.5	23.2	25.4
	**KCNG1**	150.4	92.7	351	365
	**KCNF1**	189.2	152	479.7	457.6
	**KCNJ11**	13.5	17.7	44.4	26.5
	**KCNJ6**	317.3	271.5	297.7	256.7
	**KCNQ2**	919.6	796.2	434.4	420.4
**Delayed rectifier K channels**	**KCNA5**	−4.2	0.4	99.9	84
	**KCNS1**	5.2	2.2	20.3	39.9
	**KCNH2**	25.6	32	165.6	151.9
	**KCNB1**	32.8	34.2	145.4	111.5
	**KCNB2**	25	28.7	94.5	79.1
	**KCNH2**	11.2	5.8	21	14.2
**Ca activated K channels**	**KCNN1**	6.5	0.6	48.4	28.9
	**KCNN3**	0.7	6.3	181.7	127.1
	**KCNN2**	15.8	12.3	55.6	50.1
	**KCNMB1**	62.9	60.4	44.8	72.6
**Calcium channels**	**CACNB2**	7.8	12	155.2	156.9
	**CACNG2**	2.5	11.5	123.8	115.6
	**CACNA1A**	2	10.1	64.4	62.6
	**CACNA1C**	25.9	29.3	146.9	138.8
	**CACNA1H**	122.7	87.8	268.9	220.4

Gene Set Enrichment Analysis (GSEA) was conducted to identify pathways, biological process and molecular functions that are enriched in genes differentially expressed by NSCs or dopaminergic neurons treated with amiodarone HC. In this method, all the genes are ranked according to the differential expression between two classes, and the Kolmogorov-Smirnoff test is used to determine the statistical correlation of the ranked gene list to the gene set of a given biological process, pathway or molecular function. The comparative results are then measured by a non-parametric, running sum statistic termed the enrichment score. The enrichment score significance is assessed by 1,000 permutation tests to compute the enrichment p-value. Supplementary Table 2 (Table S2) lists the pathways, biological process, and molecular functions that are significantly enriched (P value<0.05) in differentially expressed genes between drug-treated NSCs and non-treated NSCs. Supplementary Table 3 (Table S3) lists the pathways, biological process, and molecular functions that are significantly enriched (P value<0.05) in differentially expressed genes between drug-treated dopaminergic neurons and untreated populations. As shown in Supplementary Figure 2 (Figure S2), GSEA analysis revealed that cation channel activity was higher in both cohorts of untreated NSCs and dopaminergic neurons, while it was low in susceptible NSCs treated with amiodarone HCL (Fig. S2A). We noted that the tumor necrosis factor receptor 2 (TNFR2) pathway and neurogenic pathways were enriched in drug-treated NSCs (P value<0.035, Fig. S2B-E), but the two pathways were not enriched in NSCs and dopaminergic neurons prior to drug treatment. These results in their aggregate suggest that cationic channels, TNFR2-related pathways and neurogenic pathways may have important implications in the response of NSCs to amiodarone HCL drug treatment.

Based upon the GSEA results, we wanted to test our hypothesis that amiodarone HCL toxicity may act *via* specific cationic channels. We reasoned that a higher basal expression level of cation channels would render cells more susceptible to the channel blocking effect of amiodarone HCL seen in the GSEA data. Indeed, the role of amiodarone HCL in blocking multiple cation channels has been previously described [17], [18], [19], [20], [21], [22], [23], [24], [25]. To interrogate the susceptibility of both NSCs and dopaminergic neurons to amiodarone HCL-induced channel blocking, we examined differences in the expression of ion channels in both NSCs and dopaminergic neurons (Table 1). Comparison of gene expression profiles indicate that both the SLC2A1 and CLICl receptor subunit transcripts are expressed at significantly higher levels in NSCs but not in differentiated neurons, suggesting that NSCs may be more sensitive to the channel-effects of amiodarone HCL. Interestingly, published reports show that hESCs, which are intermediately affected by treatment with amiodarone HCL relative to NSCs and DA neurons (Fig. 3K), express SLC2A1 at higher levels than DA neurons, but less than the expression seen in NSCs (expression levels of 317 and 103.2 from two independent lines of BGO1, sample 131 and 122, respectively, seen in [26] ).

The TNFR2 pathway, also identified in the GSEA analysis as being selectively enriched in NSCs treated with amiodarone HCL (Fig. S2B–C), has been shown to trigger cellular apoptosis [27]. To elucidate the downstream activators of cell death in the amiodarone HCL-treated samples, we sought to examine transcription factors that were either activated or repressed four hours after exposure to the drug. To be more specific, we searched for transcription factors that were changed in NSCs after exposure to amiodarone HCl but showed no change in differentiated cells after treatment with equivalent amounts of the drug. Supplementary Table 5 lists the transcription factors. As can be seen in Table S5, amiodarone HCL treatment in NSCs significantly up regulated Fos, FosB, and DDIT3, transcription factors known to participate in TNFα receptor-mediated apoptosis through formation of the DNA-binding complex AP-1 [28], [29], [30], [31]. Notably, genes thought to induce and promote apoptosis through the intrinsic mitochondrial apoptotic pathway, such as KLF10 [32], were not altered in differentiated cells or in treated versus untreated cells. Since amiodarone HCL is known to exert its cytotoxic effect through the extrinsic, caspase-9 independent apoptotic pathway [33] our microarray results confirm that the differential cytotoxic effect seen in NSCs treated with amiodarone HCL is due to specific activation of extrinsic apoptosis pathways resulting from exposure to the drug.

**Figure 5:**
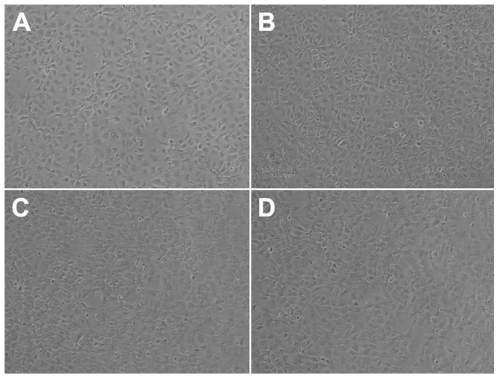
Effect of solTNFα on NSC survival. 3 concentrations of solTNFα (0.1 µM, 1 µM and 10 µM) were added to freshly seeded NSC cultures. Cells were evaluated up to 24 hours for signs of cell death. No increase in cell death relative to untreated cultures was observed in the cultures treated with solTNFα. The images taken of the cells treated with 0.1 µM of solTNFα, are representative of data obtained for all concentrations and are shown at (A) 1 hour, (B) 4 hours and (C) 24 hours post cytokine treatment. (D) Untreated cells are shown at 24 hours for comparison.

Our microarray data showed a number of genes in the TNFα pathway were highly expressed in amiodarone HCl-treated NSCs. We therefore examined whether cell death in NSCs upon amiodarone HCl exposure could be due to the activation of soluble TNFα signaling pathways. Three dosages of soluble TNFα (0.1 µM, 1 µM and 10 µM) were tested in NSC culture for 48 hours. Under these conditions we did not observe differences in cell death between treated and untreated cells (Fig. 5).

## Discussion

Our screening approach provides a new platform technology for using hESCs and purified populations of their differentiated neural derivatives to rapidly screen and identify compounds that exert specific effects on these cell types. This screening approach relies on the observable phenotype of cell death coupled with gene expression analysis to identify pathways of cell-type specific drug activity. To extend its utility, this approach can also provide clues to the molecular mechanisms that participate in stage-specific cytotoxic effects of candidate drugs. We had reasoned that because of fundamental differences in cell cycle and growth factor dependence, there would likely be drugs that were specific to one cell type versus another. Indeed, as expected in our primary screen we identified nine such compounds. Of these initial 9 candidates, 6 compounds demonstrated dose responsive toxicity exclusively in NSC populations. Interestingly, the compounds amiodarone HCL and selamectin had the most dramatic ameliorating effect on NSC survival (Fig. 2). It was surprising to us that none of these compounds were in the expected classes of anti cancer or anti-proliferative agents but instead included anti-parasitic and antiarrhythmic drugs.

We chose to further investigate one of these drugs, amiodarone HCl, which specifically killed NSCs but not dopaminergic neurons differentiated from NSCs. Amiodarone has for decades achieved clinical status as an effective class III antiarrhythmic drug in cardiac patients [34], [35]. Importantly, because it is already approved for clinical use, amiodarone HCL may have clinical applications in cell replacement therapies by selectively removing only the unwanted undifferentiated NSCs during the pre-transplant period.

In order to confirm that the cytotoxic effect seen in the amiodarone HCL-treated NSCs was specific to the activity of the drug, we first sought to determine which cellular pathways were affected in the amiodarone HCL susceptible NSC population relative to unaffected dopaminergic neurons receiving the same treatment (Fig. S2). The GSEA data revealed amiodarone HCL treated samples had significantly reduced expression of factors involved in ion channel activity. Amiodarone is known to specifically block ion channels, which suggests that the effect seen in the drug treated samples is specific to amiodarone HCL activity. To further test this, we reasoned that populations of cells with a greater basal expression of ion channel activity mediators would be most susceptible to drug treatment. Indeed, microarray data confirmed that amiodarone HCL-susceptible NSCs have significantly increased base-line expression of certain ion channels (Table 1, SLC2A1 and CLC1A). It is tantalizing to speculate that amiodarone HCl might also be toxic to other stem cell populations that demonstrate increased ion channel expression relative to their differentiated derivatives, including mesenchymal stem cells (MSCs) and endothelial precursor cells [36], thus expanding the utility of the automated screening assay described here.

Amiodarone has been shown to exert its cytotoxic effect *via* a TNF-related signaling pathway that includes caspase-8 mediated apoptosis [33]. Thus, we next wanted to determine whether our assay could detect subtle changes in TNF activity in samples treated with amiodarone HCL. Notably, downstream members of the TNFR2 pathway were significantly augmented in the amiodarone HCL-treated NSC population (Fig. S2). TNFR2 belongs to a class of membrane glycoprotein receptors that specifically bind TNFα. TNFR1 is expressed on most cell types, while TNFR2 expression is restricted to endothelial, hematopoietic and some neuronal populations [37], [38]. TNFα is a potent pro-inflammatory cytokine with two biologically active forms that are either soluble (solTNF) or membrane bound (tmTNF), and TNFR2 is preferentially activated by tmTNF [39]. It was initially thought that TNFα-mediated signaling downstream of TNFR1 results in apoptosis, while those downstream of TNFR2 induce proliferation [40]. Additional work, however, revealed that in collaboration with TNFR1, TNFα can act upon TNFR2 through a ligand passing mechanism and trigger apoptosis [40].

These published reports in their aggregate support that TNFR2 can lower the threshold of bioavailable TNFα needed to cause apoptosis through TNFR1 thus amplifying extrinsic cell death pathways. In fact, short term treatment of patients with amiodarone leads to a significant decrease in the patient's serum TNFα concentrations while paradoxically the amiodarone toxicity is exerted through TNF-mediated apoptotic pathways [41]. These observations are explained by the fact that amiodarone HCL up regulates TNFR2, and TNFR2 is more dependent on ligation with tmTNF than solTNF. To test this model, we treated amiodarone HCL-susceptible NSCs with solTNF. If amiodarone HCL toxicity is mediated through TNFR2, and TNFR2 is not sensitive to solTNF, then addition of solTNFα should not be cytotoxic to the NSCs. Indeed, three doses of solTNFα (0.1 µM, 1 µM and 10 µM) were tested in NSC culture for 48 hours and no increase in cell death relative to untreated cultures was observed (Fig. 5). This supports published reports that the addition of solTNFα to NSC cultures actually induces proliferation and differentiation [42], [43], [44]. Since TNFα is such a potent inducer of apoptosis through TNFR1 death domain signaling, and amiodarone treatment results in the down regulation of TNFα with concomitant upregulation in TNFR2 signaling in NSC alone, it is possible that amiodarone selectively kills NSCs by lowering the threshold of TNFα required to trigger apoptosis in NSCs *via* upregulation of TNFR2 pathways in NSCs and not dopaminergic neurons.

Our results support our primary goal of identifying a previously approved drug that may allow us to deplete mitotic NSCs from an otherwise differentiated population of dopaminergic neurons, thus ensuring their safety for use in transplantation. Importantly, this automated screening assay allowed us to interrogate some of the specific molecular mechanisms that may be responsible for the targeted cytotoxic effect amiodarone HCL had on NSCs and not cells differentiated from NSCs. While we do not purport to know the molecular mechanisms by which amiodarone HCL leads to the toxicity we observed in NSCs, it is notable that the results of our automated screening, including GSEA and microarray analysis, are all consistent with published literature that implicates the roles of ion channels and TNFα signaling in amiodarone-mediated cytotoxicity. This suggests that our automatic screening assay is specifically measuring the effect amiodarone HCL has on different populations of cells. Our methodology can also be easily expanded to other screens in the neural system. For example, we note that purified populations of motor neurons and oligodendrocytes are now readily available from hESCs and our screening strategy can likely be extended to these cell populations as well.

In conclusion, we describe a method using hESCs and their differentiated neural derivatives that permits the rapid screening of clinically approved drugs for compounds that can be safely used to selectively deplete progenitor cells from a differentiated cell product. Importantly, this approach is adaptable for use in a Chemistry, Manufacture and Control drug screening protocol and may have applications in identifying lineage specific reagents, thus providing additional evidence for the utility of stem cells in screening and discovery paradigms.

## Supporting Information

Table S1 Gene expression analysis
(4.55 MB XLS)

Table S2 Pathways enriched in amiodarone hcl treated NSCs
(0.21 MB DOC)

Table S3 Pathways enriched in amiodarone hcl treated dopaminergic neurons
(0.21 MB DOC)

Table S4 Controls for 96 well plates
(0.03 MB XLS)

Table S5 Transcription factors differentially expressed in NSC with and without amiodarone treatment
(0.03 MB XLS)

Figure S1 Gene expression analysis
(1.79 MB TIF)

Figure S2 GSEA analysis
(3.41 MB TIF)
