# Supplementary material for: Identification by Automated Screening of a Small Molecule that Selectively Eliminates Neural Stem Cells Derived from hESCs but Not Dopamine Neurons
Source: PLoS One. 2009 Sep 23;4(9):e7155. doi: 10.1371/journal.pone.0007155 (PMC2743191; doi:10.1371/journal.pone.0007155)
Supplement: Table S2 — Pathways enriched in amiodarone hcl treated NSCs (0.21 MB DOC) [file pone.0007155.s002.doc]

| **Activities enriched in treated NSCs** | | | |
| --- | --- | --- | --- |
| NAME | SIZE | NES | NOM p-val |
| TRANSCRIPTION_COREPRESSOR_ACTIVITY | 77 | 1.691614 | 0 |
| SERINE_HYDROLASE_ACTIVITY | 16 | 1.672039 | 0 |
| TRANSCRIPTION_REPRESSOR_ACTIVITY | 124 | 1.652917 | 0 |
| SERINE_TYPE_PEPTIDASE_ACTIVITY | 16 | 1.649524 | 0 |
| CYSTEINE_TYPE_PEPTIDASE_ACTIVITY | 42 | 1.575049 | 0 |
| ENDOPEPTIDASE_ACTIVITY | 60 | 1.465786 | 0 |
| GTPASE_REGULATOR_ACTIVITY | 106 | 1.411686 | 0 |
| PEPTIDASE_ACTIVITY | 95 | 1.400299 | 0 |
| ENZYME_REGULATOR_ACTIVITY | 234 | 1.304946 | 0.013514 |
| CYSTEINE_TYPE_ENDOPEPTIDASE_ACTIVITY | 31 | 1.621405 | 0.017857 |
| SPECIFIC_RNA_POLYMERASE_II_TRANSCRIPTION_FACTOR_ACTIVITY | 24 | 1.539421 | 0.018182 |
| PROTEIN_TYROSINE_PHOSPHATASE_ACTIVITY | 35 | 1.461157 | 0.029412 |
| PROTEASE_INHIBITOR_ACTIVITY | 20 | 1.558038 | 0.036364 |
| PHOSPHOPROTEIN_PHOSPHATASE_ACTIVITY | 56 | 1.509656 | 0.037037 |
| TRANSCRIPTION_FACTOR_BINDING | 251 | 1.247996 | 0.041667 |
| DNA_BINDING | 439 | 1.231744 | 0.049383 |
| OXIDOREDUCTASE_ACTIVITY_GO_0016705 | 17 | 1.545216 | 0.0625 |
| ENZYME_INHIBITOR_ACTIVITY | 74 | 1.362072 | 0.063492 |
| EXONUCLEASE_ACTIVITY | 17 | 1.423085 | 0.065574 |
| TRANSCRIPTION_COFACTOR_ACTIVITY | 186 | 1.217252 | 0.08 |
| DEOXYRIBONUCLEASE_ACTIVITY | 18 | 1.356687 | 0.087719 |
| PHOSPHORIC_ESTER_HYDROLASE_ACTIVITY | 105 | 1.309918 | 0.089552 |
| TRANSCRIPTION_FACTOR_ACTIVITY | 251 | 1.217464 | 0.089552 |
| PHOSPHORIC_MONOESTER_HYDROLASE_ACTIVITY | 80 | 1.310033 | 0.092105 |
| SUBSTRATE_SPECIFIC_CHANNEL_ACTIVITY | 40 | 1.299635 | 0.107143 |
| GUANYL_NUCLEOTIDE_EXCHANGE_FACTOR_ACTIVITY | 40 | 1.286659 | 0.109375 |
| PHOSPHORIC_DIESTER_HYDROLASE_ACTIVITY | 24 | 1.244372 | 0.140351 |
| RNA_POLYMERASE_II_TRANSCRIPTION_FACTOR_ACTIVITY | 126 | 1.227469 | 0.140845 |
| HYDROLASE_ACTIVITY__ACTING_ON_ESTER_BONDS | 178 | 1.18083 | 0.147059 |
| PROTEIN_COMPLEX_BINDING | 33 | 1.311813 | 0.155172 |
| GTPASE_ACTIVATOR_ACTIVITY | 48 | 1.185584 | 0.166667 |
| ION_CHANNEL_ACTIVITY | 39 | 1.254979 | 0.175439 |
| SH3_SH2_ADAPTOR_ACTIVITY | 27 | 1.235181 | 0.20339 |
| ION_TRANSMEMBRANE_TRANSPORTER_ACTIVITY | 102 | 1.185398 | 0.215385 |
| MOLECULAR_ADAPTOR_ACTIVITY | 31 | 1.189241 | 0.216667 |
| SECONDARY_ACTIVE_TRANSMEMBRANE_TRANSPORTER_ACTIVITY | 18 | 1.220784 | 0.226415 |
| ISOMERASE_ACTIVITY | 25 | 1.153866 | 0.236364 |
| ANION_TRANSMEMBRANE_TRANSPORTER_ACTIVITY | 19 | 1.216016 | 0.241379 |
| HYDROLASE_ACTIVITY__HYDROLYZING_O_GLYCOSYL_COMPOUNDS | 22 | 1.219036 | 0.245283 |
| SMALL_GTPASE_REGULATOR_ACTIVITY | 53 | 1.109262 | 0.295082 |
| PROTEIN_BINDING__BRIDGING | 37 | 1.103927 | 0.327586 |
| MOTOR_ACTIVITY | 21 | 1.104728 | 0.333333 |
| GROWTH_FACTOR_BINDING | 19 | 1.135168 | 0.351852 |
| STRUCTURAL_CONSTITUENT_OF_MUSCLE | 17 | 1.105955 | 0.363636 |
| SUBSTRATE_SPECIFIC_TRANSMEMBRANE_TRANSPORTER_ACTIVITY | 142 | 1.03954 | 0.380952 |
| METAL_ION_TRANSMEMBRANE_TRANSPORTER_ACTIVITY | 47 | 1.035591 | 0.403226 |
| STRUCTURAL_CONSTITUENT_OF_CYTOSKELETON | 32 | 1.041064 | 0.412698 |
| HYDROLASE_ACTIVITY__ACTING_ON_GLYCOSYL_BONDS | 30 | 1.048988 | 0.416667 |
| LYASE_ACTIVITY | 42 | 1.018233 | 0.424242 |
| TRANSITION_METAL_ION_BINDING | 72 | 1.036514 | 0.431034 |
| TRANSMEMBRANE_TRANSPORTER_ACTIVITY | 156 | 1.010714 | 0.434783 |
| OXIDOREDUCTASE_ACTIVITY | 180 | 0.986486 | 0.472973 |
| S_ADENOSYLMETHIONINE_DEPENDENT_METHYLTRANSFERASE_ACTIVITY | 18 | 0.997775 | 0.473684 |
| ADENYL_RIBONUCLEOTIDE_BINDING | 118 | 0.969607 | 0.476923 |
| CATION_TRANSMEMBRANE_TRANSPORTER_ACTIVITY | 81 | 1.012556 | 0.478873 |
| ZINC_ION_BINDING | 55 | 0.979693 | 0.482759 |
| ACTIN_FILAMENT_BINDING | 19 | 1.029427 | 0.508197 |
| RAS_GTPASE_ACTIVATOR_ACTIVITY | 22 | 0.981198 | 0.510204 |
| ENZYME_BINDING | 136 | 0.96839 | 0.538462 |
| NUCLEASE_ACTIVITY | 43 | 0.939313 | 0.538462 |
| TRANSMEMBRANE_RECEPTOR_PROTEIN_TYROSINE_KINASE_ACTIVITY | 30 | 0.953258 | 0.542373 |
| ADENYL_NUCLEOTIDE_BINDING | 122 | 0.948526 | 0.544118 |
| MRNA_BINDING | 17 | 1.006026 | 0.546875 |
| RHO_GTPASE_ACTIVATOR_ACTIVITY | 16 | 0.966356 | 0.565217 |
| METHYLTRANSFERASE_ACTIVITY | 29 | 0.904404 | 0.596491 |
| SMALL_GTPASE_BINDING | 29 | 0.917909 | 0.6 |
| OXIDOREDUCTASE_ACTIVITY_GO_0016616 | 34 | 0.910647 | 0.61017 |
| RNA_SPLICING_FACTOR_ACTIVITY__TRANSESTERIFICATION_MECHANISM | 17 | 0.954187 | 0.61194 |
| GTPASE_BINDING | 30 | 0.958559 | 0.612245 |
| TRANSLATION_REGULATOR_ACTIVITY | 36 | 0.921559 | 0.612903 |
| SINGLE_STRANDED_DNA_BINDING | 29 | 0.901535 | 0.616667 |
| CATION_CHANNEL_ACTIVITY | 32 | 0.902297 | 0.618182 |
| OXIDOREDUCTASE_ACTIVITY__ACTING_ON_CH_OH_GROUP_OF_DONORS | 37 | 0.865561 | 0.627119 |
| GATED_CHANNEL_ACTIVITY | 29 | 0.872611 | 0.672414 |
| SUBSTRATE_SPECIFIC_TRANSPORTER_ACTIVITY | 167 | 0.926322 | 0.676056 |
| NUCLEOTIDE_BINDING | 161 | 0.893161 | 0.686567 |
| HEMATOPOIETIN_INTERFERON_CLASS__D200_DOMAIN__CYTOKINE_RECEPTOR_ACTIVITY | 15 | 0.794707 | 0.694915 |
| PROTEIN_DOMAIN_SPECIFIC_BINDING | 45 | 0.864267 | 0.7 |
| PURINE_NUCLEOTIDE_BINDING | 150 | 0.887336 | 0.701493 |
| PURINE_RIBONUCLEOTIDE_BINDING | 146 | 0.903366 | 0.710526 |
| ATP_BINDING | 111 | 0.881342 | 0.723077 |
| MONOVALENT_INORGANIC_CATION_TRANSMEMBRANE_TRANSPORTER_ACTIVITY | 23 | 0.785686 | 0.725807 |
| TRANSLATION_FACTOR_ACTIVITY__NUCLEIC_ACID_BINDING | 34 | 0.809989 | 0.737705 |
| TRANSFERASE_ACTIVITY__TRANSFERRING_SULFUR_CONTAINING_GROUPS | 16 | 0.843519 | 0.741379 |
| ACTIVE_TRANSMEMBRANE_TRANSPORTER_ACTIVITY | 61 | 0.849457 | 0.742424 |
| SEQUENCE_SPECIFIC_DNA_BINDING | 40 | 0.824428 | 0.754717 |
| PROTEIN_TYROSINE_KINASE_ACTIVITY | 41 | 0.781789 | 0.766667 |
| PROTEIN_KINASE_ACTIVITY | 213 | 0.877212 | 0.774648 |
| TRANSFERASE_ACTIVITY__TRANSFERRING_ONE_CARBON_GROUPS | 30 | 0.795054 | 0.8 |
| GENERAL_RNA_POLYMERASE_II_TRANSCRIPTION_FACTOR_ACTIVITY | 25 | 0.772008 | 0.807018 |
| RECEPTOR_SIGNALING_PROTEIN_ACTIVITY | 58 | 0.780308 | 0.808824 |
| PROTEIN_SERINE_THREONINE_KINASE_ACTIVITY | 162 | 0.82687 | 0.811594 |
| UBIQUITIN_PROTEIN_LIGASE_ACTIVITY | 38 | 0.753572 | 0.833333 |
| STRUCTURE_SPECIFIC_DNA_BINDING | 46 | 0.761353 | 0.838235 |
| PROTEIN_KINASE_BINDING | 43 | 0.750128 | 0.842857 |
| TRANSMEMBRANE_RECEPTOR_PROTEIN_KINASE_ACTIVITY | 37 | 0.756431 | 0.846154 |
| ACTIN_BINDING | 55 | 0.745498 | 0.851852 |
| SMALL_CONJUGATING_PROTEIN_LIGASE_ACTIVITY | 40 | 0.71703 | 0.852459 |
| CALMODULIN_BINDING | 20 | 0.720394 | 0.859649 |
| ACID_AMINO_ACID_LIGASE_ACTIVITY | 45 | 0.777716 | 0.86 |
| SMALL_PROTEIN_CONJUGATING_ENZYME_ACTIVITY | 41 | 0.731379 | 0.867647 |
| TRANSFERASE_ACTIVITY__TRANSFERRING_GROUPS_OTHER_THAN_AMINO_ACYL_GROUPS | 37 | 0.675291 | 0.887097 |
| INORGANIC_CATION_TRANSMEMBRANE_TRANSPORTER_ACTIVITY | 35 | 0.680157 | 0.894737 |
| ENDONUCLEASE_ACTIVITY | 21 | 0.528946 | 0.9 |
| HYDRO_LYASE_ACTIVITY | 17 | 0.556421 | 0.907407 |
| TRANSFERASE_ACTIVITY__TRANSFERRING_ALKYL_OR_ARYL__OTHER_THAN_METHYL__GROUPS | 22 | 0.660566 | 0.913793 |
| PROTEIN_C_TERMINUS_BINDING | 58 | 0.667476 | 0.923077 |
| NUCLEAR_HORMONE_RECEPTOR_BINDING | 21 | 0.651485 | 0.928571 |
| TRANSCRIPTION_ACTIVATOR_ACTIVITY | 131 | 0.781159 | 0.942029 |
| STRUCTURAL_MOLECULE_ACTIVITY | 153 | 0.74319 | 0.942029 |
| LIGASE_ACTIVITY__FORMING_CARBON_NITROGEN_BONDS | 55 | 0.693735 | 0.948276 |
| HORMONE_RECEPTOR_BINDING | 22 | 0.620983 | 0.949153 |
| KINASE_BINDING | 49 | 0.630089 | 0.95 |
| PHOSPHATASE_REGULATOR_ACTIVITY | 20 | 0.455989 | 0.95 |
| TRANSFERASE_ACTIVITY__TRANSFERRING_GLYCOSYL_GROUPS | 73 | 0.670065 | 0.965517 |
| IDENTICAL_PROTEIN_BINDING | 212 | 0.716257 | 0.96875 |
| TRANSLATION_INITIATION_FACTOR_ACTIVITY | 22 | 0.444906 | 0.981818 |
| PROTEIN_SERINE_THREONINE_PHOSPHATASE_ACTIVITY | 18 | 0.494246 | 0.983607 |
| RIBONUCLEASE_ACTIVITY | 19 | 0.487593 | 0.983607 |
| TRANSCRIPTION_COACTIVATOR_ACTIVITY | 97 | 0.543888 | 1 |
| LIGASE_ACTIVITY | 79 | 0.467311 | 1 |
| CARBON_OXYGEN_LYASE_ACTIVITY | 21 | 0.404755 | 1 |

| **Activities enriched in untreated NSCs** | | | |
| --- | --- | --- | --- |
| NAME | SIZE | NES | NOM p-val |
| RNA_HELICASE_ACTIVITY | 23 | -1.67979 | 0 |
| ATP_DEPENDENT_RNA_HELICASE_ACTIVITY | 17 | -1.62568 | 0 |
| CALCIUM_ION_BINDING | 50 | -1.59294 | 0 |
| PHOSPHOTRANSFERASE_ACTIVITY__PHOSPHATE_GROUP_AS_ACCEPTOR | 16 | -1.66506 | 0.019231 |
| RNA_DEPENDENT_ATPASE_ACTIVITY | 18 | -1.8058 | 0.021739 |
| ATP_DEPENDENT_HELICASE_ACTIVITY | 24 | -1.49938 | 0.027778 |
| NUCLEOBASE__NUCLEOSIDE__NUCLEOTIDE_KINASE_ACTIVITY | 22 | -1.41684 | 0.047619 |
| PROTEIN_HETERODIMERIZATION_ACTIVITY | 53 | -1.3399 | 0.051282 |
| CYTOKINE_ACTIVITY | 32 | -1.38085 | 0.078947 |
| HELICASE_ACTIVITY | 46 | -1.37398 | 0.081081 |
| TRANSMEMBRANE_RECEPTOR_ACTIVITY | 150 | -1.13198 | 0.108108 |
| OXIDOREDUCTASE_ACTIVITY__ACTING_ON_THE_CH_CH_GROUP_OF_DONORS | 17 | -1.2992 | 0.131579 |
| PHOSPHOLIPID_BINDING | 31 | -1.25532 | 0.151515 |
| GROWTH_FACTOR_ACTIVITY | 23 | -1.25435 | 0.181818 |
| N_ACETYLTRANSFERASE_ACTIVITY | 17 | -1.282 | 0.195652 |
| GUANYL_NUCLEOTIDE_BINDING | 33 | -1.26525 | 0.196078 |
| LIPID_BINDING | 53 | -1.14676 | 0.209302 |
| TUBULIN_BINDING | 41 | -1.16862 | 0.219512 |
| ACETYLTRANSFERASE_ACTIVITY | 21 | -1.23949 | 0.222222 |
| ION_BINDING | 164 | -1.04371 | 0.222222 |
| N_ACYLTRANSFERASE_ACTIVITY | 19 | -1.20066 | 0.238095 |
| PYROPHOSPHATASE_ACTIVITY | 178 | -1.05291 | 0.25 |
| CYTOKINE_BINDING | 20 | -1.17419 | 0.269231 |
| DAMAGED_DNA_BINDING | 18 | -1.1681 | 0.270833 |
| G_PROTEIN_COUPLED_RECEPTOR_ACTIVITY | 47 | -1.11789 | 0.289474 |
| MAGNESIUM_ION_BINDING | 43 | -1.1144 | 0.315789 |
| PHOSPHOLIPASE_ACTIVITY | 22 | -1.11677 | 0.32 |
| DNA_DEPENDENT_ATPASE_ACTIVITY | 18 | -1.20048 | 0.326087 |
| HORMONE_ACTIVITY | 17 | -1.19559 | 0.333333 |
| CATION_BINDING | 122 | -1.03286 | 0.333333 |
| RECEPTOR_BINDING | 186 | -1.0604 | 0.357143 |
| PHOSPHOINOSITIDE_BINDING | 16 | -1.12034 | 0.375 |
| OXIDOREDUCTASE_ACTIVITY__ACTING_ON_NADH_OR_NADPH | 21 | -1.09204 | 0.378378 |
| METALLOPEPTIDASE_ACTIVITY | 23 | -1.06001 | 0.378378 |
| ATPASE_ACTIVITY__COUPLED | 73 | -1.06751 | 0.394737 |
| HYDROGEN_ION_TRANSMEMBRANE_TRANSPORTER_ACTIVITY | 20 | -1.07671 | 0.395349 |
| RHODOPSIN_LIKE_RECEPTOR_ACTIVITY | 23 | -1.04927 | 0.410256 |
| CHROMATIN_BINDING | 28 | -1.01492 | 0.413043 |
| PEPTIDE_BINDING | 35 | -0.93863 | 0.416667 |
| LIPASE_ACTIVITY | 22 | -1.05475 | 0.428571 |
| RECEPTOR_ACTIVITY | 228 | -1.04375 | 0.428571 |
| TRANSFERASE_ACTIVITY__TRANSFERRING_PHOSPHORUS_CONTAINING_GROUPS | 321 | -1.00233 | 0.434783 |
| CARBOHYDRATE_BINDING | 29 | -0.97286 | 0.452381 |
| HEPARIN_BINDING | 18 | -1.01491 | 0.461538 |
| ENZYME_ACTIVATOR_ACTIVITY | 90 | -1.02383 | 0.470588 |
| PROTEIN_DIMERIZATION_ACTIVITY | 119 | -1.01681 | 0.475 |
| KINASE_ACTIVITY | 280 | -0.9611 | 0.47619 |
| KINASE_REGULATOR_ACTIVITY | 31 | -0.92865 | 0.52381 |
| COFACTOR_BINDING | 17 | -0.93262 | 0.545455 |
| HYDROLASE_ACTIVITY__ACTING_ON_ACID_ANHYDRIDES | 180 | -0.99065 | 0.548387 |
| PATTERN_BINDING | 22 | -0.89988 | 0.555556 |
| GLYCOSAMINOGLYCAN_BINDING | 22 | -0.91273 | 0.560976 |
| RAS_GTPASE_BINDING | 21 | -0.95668 | 0.564103 |
| AMINE_TRANSMEMBRANE_TRANSPORTER_ACTIVITY | 18 | -0.90019 | 0.575 |
| UDP_GLYCOSYLTRANSFERASE_ACTIVITY | 23 | -0.94593 | 0.589744 |
| POLYSACCHARIDE_BINDING | 22 | -0.96752 | 0.604651 |
| AMINO_ACID_TRANSMEMBRANE_TRANSPORTER_ACTIVITY | 16 | -0.94549 | 0.613636 |
| GTP_BINDING | 32 | -0.96951 | 0.622222 |
| PROTEIN_KINASE_REGULATOR_ACTIVITY | 27 | -0.88093 | 0.636364 |
| CARBOXYLIC_ACID_TRANSMEMBRANE_TRANSPORTER_ACTIVITY | 20 | -0.83731 | 0.641026 |
| EXOPEPTIDASE_ACTIVITY | 18 | -0.91499 | 0.642857 |
| SIGNAL_SEQUENCE_BINDING | 15 | -0.82865 | 0.642857 |
| KINASE_INHIBITOR_ACTIVITY | 16 | -0.80018 | 0.682927 |
| PHOSPHOTRANSFERASE_ACTIVITY__ALCOHOL_GROUP_AS_ACCEPTOR | 251 | -0.92896 | 0.7 |
| TRANSFERASE_ACTIVITY__TRANSFERRING_ACYL_GROUPS | 43 | -0.91463 | 0.717949 |
| TRANSFERASE_ACTIVITY__TRANSFERRING_HEXOSYL_GROUPS | 52 | -0.88142 | 0.727273 |
| ORGANIC_ACID_TRANSMEMBRANE_TRANSPORTER_ACTIVITY | 20 | -0.81932 | 0.763158 |
| GTPASE_ACTIVITY | 74 | -0.81096 | 0.763158 |
| PROTEIN_KINASE_INHIBITOR_ACTIVITY | 16 | -0.78055 | 0.764706 |
| ATPASE_ACTIVITY | 91 | -0.82526 | 0.782609 |
| RECEPTOR_SIGNALING_PROTEIN_SERINE_THREONINE_KINASE_ACTIVITY | 26 | -0.79537 | 0.782609 |
| HYDROLASE_ACTIVITY__ACTING_ON_CARBON_NITROGEN__BUT_NOT_PEPTIDE__BONDS | 28 | -0.78229 | 0.790698 |
| PROTEIN_HOMODIMERIZATION_ACTIVITY | 75 | -0.80097 | 0.8 |
| NUCLEOSIDE_TRIPHOSPHATASE_ACTIVITY | 166 | -0.89946 | 0.827586 |
| MICROTUBULE_BINDING | 29 | -0.71432 | 0.864865 |
| ATPASE_ACTIVITY__COUPLED_TO_TRANSMEMBRANE_MOVEMENT_OF_IONS | 15 | -0.54303 | 0.880952 |
| UNFOLDED_PROTEIN_BINDING | 37 | -0.65076 | 0.882353 |
| NUCLEOTIDYLTRANSFERASE_ACTIVITY | 37 | -0.6782 | 0.921053 |
| STRUCTURAL_CONSTITUENT_OF_RIBOSOME | 69 | -0.63433 | 0.939394 |
| CYTOSKELETAL_PROTEIN_BINDING | 117 | -0.78998 | 0.944444 |
| PROTEIN_N_TERMINUS_BINDING | 29 | -0.55418 | 0.944444 |
| HYDROLASE_ACTIVITY__ACTING_ON_ACID_ANHYDRIDES__CATALYZING_TRANSMEMBRANE_MOVEMENT_OF_SUBSTANCES | 25 | -0.40967 | 0.975 |
| PRIMARY_ACTIVE_TRANSMEMBRANE_TRANSPORTER_ACTIVITY | 26 | -0.38584 | 0.97619 |
| DNA_HELICASE_ACTIVITY | 21 | -0.55417 | 0.977778 |
| ELECTRON_CARRIER_ACTIVITY | 57 | -0.63073 | 0.978261 |
| RNA_BINDING | 206 | -0.66586 | 1 |
| DOUBLE_STRANDED_DNA_BINDING | 28 | -0.4584 | 1 |
| ATPASE_ACTIVITY__COUPLED_TO_MOVEMENT_OF_SUBSTANCES | 26 | -0.37429 | 1 |
