# Supplementary material for: Identification by Automated Screening of a Small Molecule that Selectively Eliminates Neural Stem Cells Derived from hESCs but Not Dopamine Neurons
Source: PLoS One. 2009 Sep 23;4(9):e7155. doi: 10.1371/journal.pone.0007155 (PMC2743191; doi:10.1371/journal.pone.0007155)
Supplement: Table S3 — Pathways enriched in amiodarone hcl treated dopaminergic neurons (0.21 MB DOC) [file pone.0007155.s003.doc]

| **Activities enriched in treated DA neurons** | | | |
| --- | --- | --- | --- |
| NAME | SIZE | NES | NOM p-val |
| SECONDARY_ACTIVE_TRANSMEMBRANE_TRANSPORTER_ACTIVITY | 21 | 1.632949 | 0 |
| PROTEIN_SERINE_THREONINE_KINASE_ACTIVITY | 164 | 1.386265 | 0.027778 |
| SEQUENCE_SPECIFIC_DNA_BINDING | 37 | 1.388153 | 0.056338 |
| PHOSPHOTRANSFERASE_ACTIVITY__ALCOHOL_GROUP_AS_ACCEPTOR | 249 | 1.237356 | 0.059524 |
| ANION_TRANSMEMBRANE_TRANSPORTER_ACTIVITY | 25 | 1.432292 | 0.065574 |
| LIPASE_ACTIVITY | 21 | 1.421672 | 0.067797 |
| ACTIVE_TRANSMEMBRANE_TRANSPORTER_ACTIVITY | 71 | 1.375974 | 0.082192 |
| MONOVALENT_INORGANIC_CATION_TRANSMEMBRANE_TRANSPORTER_ACTIVITY | 25 | 1.3181 | 0.122807 |
| RECEPTOR_SIGNALING_PROTEIN_SERINE_THREONINE_KINASE_ACTIVITY | 29 | 1.268038 | 0.157895 |
| PROTEIN_KINASE_ACTIVITY | 214 | 1.195546 | 0.176471 |
| PEPTIDE_BINDING | 41 | 1.226343 | 0.181818 |
| STRUCTURAL_CONSTITUENT_OF_MUSCLE | 21 | 1.208285 | 0.207547 |
| STRUCTURE_SPECIFIC_DNA_BINDING | 44 | 1.183457 | 0.230769 |
| RECEPTOR_SIGNALING_PROTEIN_ACTIVITY | 59 | 1.148881 | 0.236842 |
| LIGASE_ACTIVITY__FORMING_CARBON_NITROGEN_BONDS | 58 | 1.126519 | 0.242857 |
| SMALL_CONJUGATING_PROTEIN_LIGASE_ACTIVITY | 43 | 1.14368 | 0.243243 |
| PHOSPHORIC_DIESTER_HYDROLASE_ACTIVITY | 26 | 1.182993 | 0.25 |
| RHODOPSIN_LIKE_RECEPTOR_ACTIVITY | 28 | 1.14436 | 0.25 |
| LIGASE_ACTIVITY | 83 | 1.139904 | 0.25 |
| SMALL_PROTEIN_CONJUGATING_ENZYME_ACTIVITY | 44 | 1.146143 | 0.257576 |
| DEOXYRIBONUCLEASE_ACTIVITY | 17 | 1.315043 | 0.258621 |
| ACID_AMINO_ACID_LIGASE_ACTIVITY | 48 | 1.17974 | 0.28 |
| UBIQUITIN_PROTEIN_LIGASE_ACTIVITY | 41 | 1.138988 | 0.301587 |
| ATP_BINDING | 114 | 1.076941 | 0.318841 |
| OXIDOREDUCTASE_ACTIVITY_GO_0016616 | 34 | 1.058256 | 0.353846 |
| ATPASE_ACTIVITY__COUPLED_TO_MOVEMENT_OF_SUBSTANCES | 29 | 1.065411 | 0.355932 |
| DNA_BINDING | 431 | 1.034326 | 0.359551 |
| NUCLEASE_ACTIVITY | 40 | 1.071979 | 0.360656 |
| PHOSPHOLIPASE_ACTIVITY | 20 | 1.124752 | 0.363636 |
| UDP_GLYCOSYLTRANSFERASE_ACTIVITY | 27 | 1.093145 | 0.366667 |
| HYDROGEN_ION_TRANSMEMBRANE_TRANSPORTER_ACTIVITY | 21 | 1.077725 | 0.366667 |
| KINASE_ACTIVITY | 277 | 1.036083 | 0.367816 |
| RNA_POLYMERASE_II_TRANSCRIPTION_FACTOR_ACTIVITY | 124 | 1.041575 | 0.371429 |
| HYDROLASE_ACTIVITY__ACTING_ON_ACID_ANHYDRIDES__CATALYZING_TRANSMEMBRANE_MOVEMENT_OF_SUBSTANCES | 28 | 1.094199 | 0.376812 |
| DOUBLE_STRANDED_DNA_BINDING | 26 | 1.071692 | 0.37931 |
| ENZYME_ACTIVATOR_ACTIVITY | 92 | 1.048951 | 0.382353 |
| TRANSMEMBRANE_RECEPTOR_ACTIVITY | 170 | 1.054918 | 0.382716 |
| INORGANIC_CATION_TRANSMEMBRANE_TRANSPORTER_ACTIVITY | 39 | 1.065105 | 0.40625 |
| ENZYME_INHIBITOR_ACTIVITY | 70 | 1.052384 | 0.409091 |
| TRANSFERASE_ACTIVITY__TRANSFERRING_GROUPS_OTHER_THAN_AMINO_ACYL_GROUPS | 34 | 1.050073 | 0.415385 |
| ENDONUCLEASE_ACTIVITY | 20 | 0.985867 | 0.419355 |
| CYTOKINE_ACTIVITY | 30 | 1.062567 | 0.421875 |
| OXIDOREDUCTASE_ACTIVITY__ACTING_ON_CH_OH_GROUP_OF_DONORS | 37 | 1.053572 | 0.424242 |
| OXIDOREDUCTASE_ACTIVITY__ACTING_ON_THE_CH_CH_GROUP_OF_DONORS | 18 | 1.027969 | 0.428571 |
| PRIMARY_ACTIVE_TRANSMEMBRANE_TRANSPORTER_ACTIVITY | 29 | 1.055886 | 0.430556 |
| GTPASE_ACTIVITY | 79 | 1.01517 | 0.442857 |
| TRANSFERASE_ACTIVITY__TRANSFERRING_ACYL_GROUPS | 39 | 1.002919 | 0.451613 |
| DAMAGED_DNA_BINDING | 17 | 1.035788 | 0.45283 |
| PHOSPHATASE_REGULATOR_ACTIVITY | 21 | 1.041847 | 0.467742 |
| N_ACETYLTRANSFERASE_ACTIVITY | 15 | 1.019038 | 0.473684 |
| STRUCTURAL_CONSTITUENT_OF_CYTOSKELETON | 34 | 0.970783 | 0.482759 |
| ADENYL_RIBONUCLEOTIDE_BINDING | 120 | 0.984786 | 0.506667 |
| G_PROTEIN_COUPLED_RECEPTOR_ACTIVITY | 55 | 0.985169 | 0.514706 |
| PROTEASE_INHIBITOR_ACTIVITY | 20 | 0.98644 | 0.516667 |
| TRANSMEMBRANE_RECEPTOR_PROTEIN_KINASE_ACTIVITY | 33 | 0.964975 | 0.516667 |
| N_ACYLTRANSFERASE_ACTIVITY | 17 | 0.988866 | 0.519231 |
| ENZYME_REGULATOR_ACTIVITY | 228 | 0.979318 | 0.520548 |
| HORMONE_ACTIVITY | 18 | 0.97489 | 0.523077 |
| GENERAL_RNA_POLYMERASE_II_TRANSCRIPTION_FACTOR_ACTIVITY | 24 | 0.923838 | 0.530612 |
| PYROPHOSPHATASE_ACTIVITY | 183 | 0.953558 | 0.54321 |
| RECEPTOR_ACTIVITY | 256 | 0.97382 | 0.5625 |
| PURINE_RIBONUCLEOTIDE_BINDING | 149 | 0.957684 | 0.5625 |
| TRANSFERASE_ACTIVITY__TRANSFERRING_SULFUR_CONTAINING_GROUPS | 21 | 0.940302 | 0.566667 |
| MAGNESIUM_ION_BINDING | 43 | 0.940149 | 0.567568 |
| INTEGRIN_BINDING | 17 | 0.914059 | 0.571429 |
| SUBSTRATE_SPECIFIC_TRANSPORTER_ACTIVITY | 211 | 0.92899 | 0.573171 |
| ACETYLTRANSFERASE_ACTIVITY | 19 | 0.947398 | 0.6 |
| SINGLE_STRANDED_DNA_BINDING | 26 | 0.953332 | 0.605634 |
| PROTEIN_HOMODIMERIZATION_ACTIVITY | 82 | 0.881709 | 0.621212 |
| PROTEIN_DOMAIN_SPECIFIC_BINDING | 52 | 0.935722 | 0.628571 |
| LIPID_TRANSPORTER_ACTIVITY | 15 | 0.919447 | 0.62963 |
| IDENTICAL_PROTEIN_BINDING | 217 | 0.952644 | 0.630137 |
| PROTEIN_DIMERIZATION_ACTIVITY | 128 | 0.924809 | 0.636364 |
| ION_TRANSMEMBRANE_TRANSPORTER_ACTIVITY | 139 | 0.927608 | 0.64 |
| TRANSFERASE_ACTIVITY__TRANSFERRING_HEXOSYL_GROUPS | 58 | 0.926571 | 0.642857 |
| NUCLEOTIDE_BINDING | 164 | 0.932434 | 0.643836 |
| TRANSFERASE_ACTIVITY__TRANSFERRING_PHOSPHORUS_CONTAINING_GROUPS | 317 | 0.94993 | 0.64557 |
| ZINC_ION_BINDING | 52 | 0.89596 | 0.647059 |
| TRANSLATION_REGULATOR_ACTIVITY | 35 | 0.876409 | 0.661017 |
| TRANSLATION_FACTOR_ACTIVITY__NUCLEIC_ACID_BINDING | 33 | 0.845424 | 0.681159 |
| CARBOHYDRATE_BINDING | 28 | 0.888154 | 0.688525 |
| ADENYL_NUCLEOTIDE_BINDING | 125 | 0.884667 | 0.694118 |
| MICROTUBULE_BINDING | 30 | 0.812262 | 0.696429 |
| SUBSTRATE_SPECIFIC_TRANSMEMBRANE_TRANSPORTER_ACTIVITY | 182 | 0.907061 | 0.697368 |
| TRANSCRIPTION_COFACTOR_ACTIVITY | 186 | 0.877451 | 0.7 |
| TRANSCRIPTION_COACTIVATOR_ACTIVITY | 98 | 0.877727 | 0.708333 |
| SULFOTRANSFERASE_ACTIVITY | 17 | 0.88001 | 0.719298 |
| ENDOPEPTIDASE_ACTIVITY | 66 | 0.813589 | 0.720588 |
| METALLOPEPTIDASE_ACTIVITY | 21 | 0.873137 | 0.733333 |
| NEUROTRANSMITTER_BINDING | 15 | 0.789043 | 0.754717 |
| NEUROTRANSMITTER_RECEPTOR_ACTIVITY | 15 | 0.797205 | 0.757576 |
| LYASE_ACTIVITY | 47 | 0.864626 | 0.758065 |
| TRANSMEMBRANE_TRANSPORTER_ACTIVITY | 196 | 0.898466 | 0.759494 |
| TRANSCRIPTION_REPRESSOR_ACTIVITY | 122 | 0.855519 | 0.76 |
| NUCLEOSIDE_TRIPHOSPHATASE_ACTIVITY | 173 | 0.873411 | 0.761905 |
| PURINE_NUCLEOTIDE_BINDING | 154 | 0.861007 | 0.763158 |
| HYDROLASE_ACTIVITY__ACTING_ON_ACID_ANHYDRIDES | 185 | 0.868824 | 0.770115 |
| MOTOR_ACTIVITY | 22 | 0.771596 | 0.77193 |
| CALCIUM_CHANNEL_ACTIVITY | 18 | 0.753383 | 0.781818 |
| HYDRO_LYASE_ACTIVITY | 19 | 0.773039 | 0.792453 |
| CALCIUM_ION_BINDING | 56 | 0.791332 | 0.805556 |
| HYDROLASE_ACTIVITY__ACTING_ON_CARBON_NITROGEN__BUT_NOT_PEPTIDE__BONDS__IN_LINEAR_AMIDES | 15 | 0.666303 | 0.826923 |
| TRANSMEMBRANE_RECEPTOR_PROTEIN_TYROSINE_KINASE_ACTIVITY | 27 | 0.785587 | 0.828947 |
| ION_BINDING | 166 | 0.812988 | 0.833333 |
| MOLECULAR_ADAPTOR_ACTIVITY | 35 | 0.768201 | 0.84507 |
| GTP_BINDING | 33 | 0.769023 | 0.848485 |
| GUANYL_NUCLEOTIDE_EXCHANGE_FACTOR_ACTIVITY | 36 | 0.770802 | 0.857143 |
| CARBON_OXYGEN_LYASE_ACTIVITY | 23 | 0.740198 | 0.86 |
| TRANSFERASE_ACTIVITY__TRANSFERRING_GLYCOSYL_GROUPS | 79 | 0.829738 | 0.864865 |
| CATION_BINDING | 125 | 0.782515 | 0.894737 |
| PROTEIN_N_TERMINUS_BINDING | 30 | 0.716727 | 0.910714 |
| CALMODULIN_BINDING | 19 | 0.691734 | 0.910714 |
| GUANYL_NUCLEOTIDE_BINDING | 34 | 0.720005 | 0.913044 |
| HYDROLASE_ACTIVITY__ACTING_ON_ESTER_BONDS | 185 | 0.77466 | 0.924051 |
| TRANSCRIPTION_COREPRESSOR_ACTIVITY | 76 | 0.695835 | 0.942857 |
| ATPASE_ACTIVITY__COUPLED_TO_TRANSMEMBRANE_MOVEMENT_OF_IONS | 17 | 0.593549 | 0.944444 |
| UNFOLDED_PROTEIN_BINDING | 38 | 0.625776 | 0.955224 |
| STRUCTURAL_MOLECULE_ACTIVITY | 157 | 0.732434 | 0.963415 |
| TRANSFERASE_ACTIVITY__TRANSFERRING_ALKYL_OR_ARYL__OTHER_THAN_METHYL__GROUPS | 21 | 0.628434 | 0.968254 |
| ACTIN_FILAMENT_BINDING | 19 | 0.313769 | 0.983871 |
| MRNA_BINDING | 18 | 0.408682 | 0.984375 |
| TRANSCRIPTION_FACTOR_BINDING | 252 | 0.742013 | 0.987013 |
| PHOSPHORIC_ESTER_HYDROLASE_ACTIVITY | 113 | 0.695887 | 0.9875 |
| ATPASE_ACTIVITY | 93 | 0.65281 | 1 |
| RNA_BINDING | 209 | 0.440631 | 1 |
| TRANSLATION_INITIATION_FACTOR_ACTIVITY | 22 | 0.331139 | 1 |

| **Activities enriched in untreated DA neurons** | | | |
| --- | --- | --- | --- |
| NAME | SIZE | NES | NOM p-val |
| GTPASE_BINDING | 29 | -1.61975 | 0 |
| HYDROLASE_ACTIVITY__HYDROLYZING_O_GLYCOSYL_COMPOUNDS | 25 | -1.47947 | 0 |
| RAS_GTPASE_BINDING | 21 | -1.58473 | 0.02 |
| CATION_CHANNEL_ACTIVITY | 57 | -1.39352 | 0.027778 |
| SMALL_GTPASE_BINDING | 28 | -1.4967 | 0.047619 |
| GATED_CHANNEL_ACTIVITY | 54 | -1.49125 | 0.051282 |
| ION_CHANNEL_ACTIVITY | 66 | -1.19958 | 0.060606 |
| HYDROLASE_ACTIVITY__ACTING_ON_GLYCOSYL_BONDS | 33 | -1.41401 | 0.08 |
| NUCLEOBASE__NUCLEOSIDE__NUCLEOTIDE_KINASE_ACTIVITY | 22 | -1.36784 | 0.081081 |
| VOLTAGE_GATED_CHANNEL_ACTIVITY | 31 | -1.28394 | 0.085714 |
| KINASE_BINDING | 51 | -1.31564 | 0.1 |
| EXOPEPTIDASE_ACTIVITY | 18 | -1.3223 | 0.105263 |
| POTASSIUM_CHANNEL_ACTIVITY | 23 | -1.32529 | 0.111111 |
| METAL_ION_TRANSMEMBRANE_TRANSPORTER_ACTIVITY | 76 | -1.26886 | 0.111111 |
| SUBSTRATE_SPECIFIC_CHANNEL_ACTIVITY | 68 | -1.22108 | 0.129032 |
| CHROMATIN_BINDING | 28 | -1.23921 | 0.142857 |
| GTPASE_ACTIVATOR_ACTIVITY | 48 | -1.22185 | 0.157895 |
| PHOSPHOPROTEIN_PHOSPHATASE_ACTIVITY | 63 | -1.15038 | 0.162162 |
| ELECTRON_CARRIER_ACTIVITY | 55 | -1.20425 | 0.166667 |
| VOLTAGE_GATED_CATION_CHANNEL_ACTIVITY | 29 | -1.17396 | 0.166667 |
| TRANSITION_METAL_ION_BINDING | 69 | -1.09776 | 0.171429 |
| PROTEIN_C_TERMINUS_BINDING | 60 | -1.19544 | 0.212121 |
| HELICASE_ACTIVITY | 46 | -1.18729 | 0.216216 |
| PROTEIN_COMPLEX_BINDING | 35 | -1.13316 | 0.222222 |
| ENZYME_BINDING | 136 | -1.04 | 0.233333 |
| RAS_GTPASE_ACTIVATOR_ACTIVITY | 23 | -1.14374 | 0.243902 |
| S_ADENOSYLMETHIONINE_DEPENDENT_METHYLTRANSFERASE_ACTIVITY | 18 | -1.2358 | 0.25 |
| LIGAND_DEPENDENT_NUCLEAR_RECEPTOR_ACTIVITY | 18 | -1.17725 | 0.25641 |
| PROTEIN_KINASE_BINDING | 44 | -1.11558 | 0.258065 |
| LIGAND_GATED_CHANNEL_ACTIVITY | 17 | -1.23641 | 0.263158 |
| CATION_TRANSMEMBRANE_TRANSPORTER_ACTIVITY | 111 | -1.08038 | 0.291667 |
| RHO_GTPASE_ACTIVATOR_ACTIVITY | 15 | -1.15992 | 0.297297 |
| RNA_DEPENDENT_ATPASE_ACTIVITY | 17 | -1.10955 | 0.318182 |
| DNA_HELICASE_ACTIVITY | 22 | -1.12245 | 0.319149 |
| ATP_DEPENDENT_HELICASE_ACTIVITY | 23 | -1.13329 | 0.324324 |
| METHYLTRANSFERASE_ACTIVITY | 29 | -1.089 | 0.340909 |
| AUXILIARY_TRANSPORT_PROTEIN_ACTIVITY | 15 | -1.14107 | 0.347826 |
| TRANSCRIPTION_FACTOR_ACTIVITY | 246 | -1.02235 | 0.35 |
| RNA_HELICASE_ACTIVITY | 22 | -1.05343 | 0.365854 |
| GROWTH_FACTOR_ACTIVITY | 25 | -1.07927 | 0.367347 |
| SMALL_GTPASE_REGULATOR_ACTIVITY | 54 | -1.03771 | 0.387097 |
| PHOSPHOTRANSFERASE_ACTIVITY__PHOSPHATE_GROUP_AS_ACCEPTOR | 16 | -1.05813 | 0.431818 |
| PROTEIN_TYROSINE_PHOSPHATASE_ACTIVITY | 39 | -1.01857 | 0.432432 |
| TUBULIN_BINDING | 40 | -0.98943 | 0.444444 |
| PEPTIDASE_ACTIVITY | 101 | -0.9811 | 0.444444 |
| CYTOKINE_BINDING | 19 | -0.96435 | 0.459459 |
| GTPASE_REGULATOR_ACTIVITY | 102 | -0.96851 | 0.461538 |
| ACTIN_BINDING | 60 | -1.00722 | 0.464286 |
| TRANSFERASE_ACTIVITY__TRANSFERRING_ONE_CARBON_GROUPS | 30 | -0.99648 | 0.466667 |
| RECEPTOR_BINDING | 189 | -0.98651 | 0.47619 |
| PROTEIN_SERINE_THREONINE_PHOSPHATASE_ACTIVITY | 18 | -0.99073 | 0.48718 |
| PROTEIN_BINDING__BRIDGING | 40 | -0.9268 | 0.5 |
| POLYSACCHARIDE_BINDING | 19 | -0.92306 | 0.5 |
| ATP_DEPENDENT_RNA_HELICASE_ACTIVITY | 16 | -1.02238 | 0.52381 |
| SH3_SH2_ADAPTOR_ACTIVITY | 30 | -0.87549 | 0.542857 |
| PATTERN_BINDING | 19 | -0.94385 | 0.567568 |
| GLYCOSAMINOGLYCAN_BINDING | 19 | -0.90975 | 0.571429 |
| PHOSPHORIC_MONOESTER_HYDROLASE_ACTIVITY | 86 | -0.9735 | 0.612903 |
| HYDROLASE_ACTIVITY__ACTING_ON_CARBON_NITROGEN__BUT_NOT_PEPTIDE__BONDS | 29 | -0.85011 | 0.634146 |
| COFACTOR_BINDING | 16 | -0.85706 | 0.636364 |
| PROTEIN_TYROSINE_KINASE_ACTIVITY | 39 | -0.83815 | 0.657143 |
| OXIDOREDUCTASE_ACTIVITY_GO_0016705 | 19 | -0.86064 | 0.675676 |
| GROWTH_FACTOR_BINDING | 17 | -0.83417 | 0.705882 |
| SIGNAL_SEQUENCE_BINDING | 15 | -0.81856 | 0.707317 |
| CYSTEINE_TYPE_PEPTIDASE_ACTIVITY | 43 | -0.84396 | 0.714286 |
| CYTOSKELETAL_PROTEIN_BINDING | 122 | -0.90039 | 0.730769 |
| DNA_DEPENDENT_ATPASE_ACTIVITY | 18 | -0.83316 | 0.783784 |
| OXIDOREDUCTASE_ACTIVITY | 186 | -0.91477 | 0.818182 |
| CYSTEINE_TYPE_ENDOPEPTIDASE_ACTIVITY | 32 | -0.8093 | 0.818182 |
| ORGANIC_ACID_TRANSMEMBRANE_TRANSPORTER_ACTIVITY | 24 | -0.72342 | 0.833333 |
| LIPID_BINDING | 55 | -0.79643 | 0.852941 |
| RIBONUCLEASE_ACTIVITY | 17 | -0.61729 | 0.875 |
| PROTEIN_KINASE_REGULATOR_ACTIVITY | 25 | -0.73437 | 0.885714 |
| RNA_SPLICING_FACTOR_ACTIVITY__TRANSESTERIFICATION_MECHANISM | 17 | -0.56751 | 0.891892 |
| SPECIFIC_RNA_POLYMERASE_II_TRANSCRIPTION_FACTOR_ACTIVITY | 23 | -0.64118 | 0.904762 |
| NUCLEAR_HORMONE_RECEPTOR_BINDING | 20 | -0.49296 | 0.904762 |
| TRANSCRIPTION_ACTIVATOR_ACTIVITY | 130 | -0.84114 | 0.90625 |
| ISOMERASE_ACTIVITY | 28 | -0.60478 | 0.90625 |
| SERINE_TYPE_ENDOPEPTIDASE_ACTIVITY | 18 | -0.65879 | 0.911111 |
| KINASE_REGULATOR_ACTIVITY | 30 | -0.69893 | 0.911765 |
| PHOSPHOLIPID_BINDING | 31 | -0.68686 | 0.914894 |
| ATPASE_ACTIVITY__COUPLED | 74 | -0.7071 | 0.925926 |
| CARBOXYLIC_ACID_TRANSMEMBRANE_TRANSPORTER_ACTIVITY | 24 | -0.69599 | 0.947368 |
| PHOSPHOINOSITIDE_BINDING | 16 | -0.56037 | 0.953488 |
| SERINE_HYDROLASE_ACTIVITY | 21 | -0.63038 | 0.955556 |
| AMINO_ACID_TRANSMEMBRANE_TRANSPORTER_ACTIVITY | 20 | -0.49382 | 0.955556 |
| HORMONE_RECEPTOR_BINDING | 21 | -0.47737 | 0.969697 |
| PROTEIN_HETERODIMERIZATION_ACTIVITY | 56 | -0.79087 | 0.971429 |
| OXIDOREDUCTASE_ACTIVITY__ACTING_ON_NADH_OR_NADPH | 21 | -0.49945 | 0.975 |
| SERINE_TYPE_PEPTIDASE_ACTIVITY | 21 | -0.65289 | 0.97561 |
| NUCLEOTIDYLTRANSFERASE_ACTIVITY | 34 | -0.49704 | 1 |
| AMINE_TRANSMEMBRANE_TRANSPORTER_ACTIVITY | 22 | -0.41078 | 1 |
| STRUCTURAL_CONSTITUENT_OF_RIBOSOME | 66 | -0.26757 | 1 |
