# Supplementary figures and images for: Identification by Automated Screening of a Small Molecule that Selectively Eliminates Neural Stem Cells Derived from hESCs but Not Dopamine Neurons
Source: PLoS One. 2009 Sep 23;4(9):e7155. doi: 10.1371/journal.pone.0007155 (PMC2743191; doi:10.1371/journal.pone.0007155)

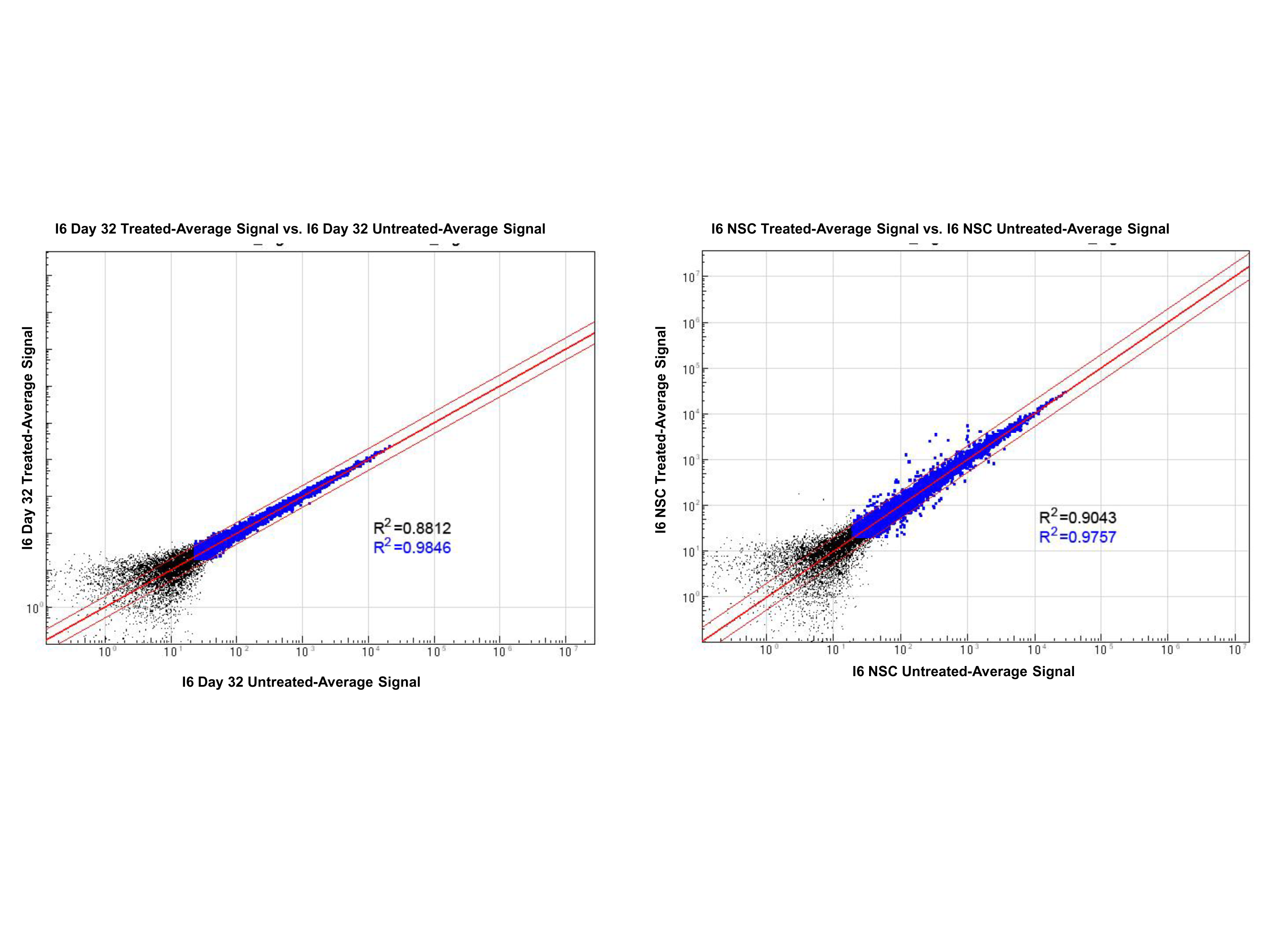

Supplement: Figure S1 — Gene expression analysis (1.79 MB TIF) [file pone.0007155.s006.tif]

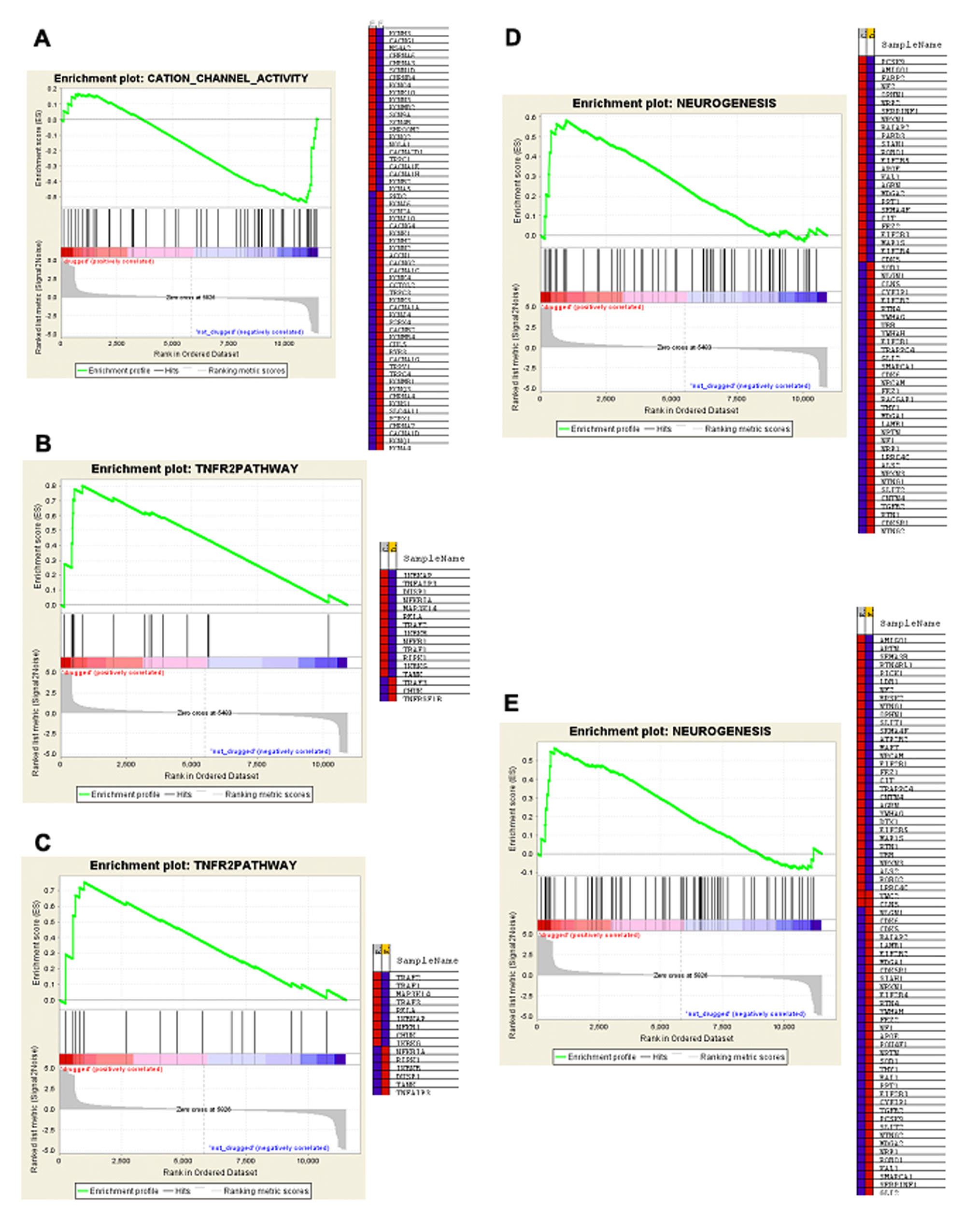

Supplement: Figure S2 — GSEA analysis (3.41 MB TIF) [file pone.0007155.s007.tif]
